# Interruption and Defaulting of Multidrug Therapy against Leprosy: Population-Based Study in Brazil's Savannah Region

**DOI:** 10.1371/journal.pntd.0001031

**Published:** 2011-05-03

**Authors:** Jorg Heukelbach, Olga André Chichava, Alexcian Rodrigues de Oliveira, Kathrin Häfner, Friederike Walther, Carlos Henrique Morais de Alencar, Alberto Novaes Ramos, Adriana Cavalcante Ferreira, Liana Ariza

**Affiliations:** 1 Department of Community Health, School of Medicine, Federal University of Ceará, Fortaleza, Brazil; 2 Anton Breinl Centre for Public Health and Tropical Medicine, School of Public Health, Tropical Medicine and Rehabilitation Sciences, James Cook University, Townsville, Australia; 3 School of Medicine, University of Düsseldorf, Düsseldorf, Germany; 4 School of Medicine, University of Cologne, Cologne, Germany; 5 State Leprosy Control Program, State Health Secretariat of Tocantins, Palmas, Brazil; Emory University, United States of America

## Abstract

**Background:**

Low adherence to multidrug therapy against leprosy (MDT) is still an important obstacle of disease control, and may lead to remaining sources of infection, incomplete cure, irreversible complications, and multidrug resistance.

**Methodology/Principal Finding:**

We performed a population-based study in 78 municipalities in Tocantins State, central Brazil, and applied structured questionnaires on leprosy-affected individuals. We used two outcomes for assessment of risk factors: defaulting (not presenting to health care center for supervised treatment for >12 months); and interruption of MDT. In total, 28/936 (3.0%) patients defaulted, and 147/806 (18.2%) interrupted MDT. Defaulting was significantly associated with: low number of rooms per household (OR = 3.43; 0.98–9.69; p = 0.03); moving to another residence after diagnosis (OR = 2.90; 0.95–5.28; p = 0.04); and low family income (OR = 2.42; 1.02–5.63: p = 0.04). Interruption of treatment was associated with: low number of rooms per household (OR = 1.95; 0.98–3.70; p = 0.04); difficulty in swallowing MDT drugs (OR = 1.66; 1.03–2.63; p = 0.02); temporal non-availability of MDT at the health center (OR = 1.67; 1.11–2.46; p = 0.01); and moving to another residence (OR = 1.58; 95% confidence interval: 1.03–2.40; p = 0.03). Logistic regression identified temporal non-availability of MDT as an independent risk factor for treatment interruption (adjusted OR = 1.56; 1.05–2.33; p = 0.03), and residence size as a protective factor (adjusted OR = 0.89 per additional number of rooms; 0.80–0.99; p = 0.03). Residence size was also independently associated with defaulting (adjusted OR = 0.67; 0.52–0.88; p = 0.003).

**Conclusions:**

Defaulting and interruption of MDT are associated with some poverty-related variables such as family income, household size, and migration. Intermittent problems of drug supply need to be resolved, mainly on the municipality level. MDT producers should consider oral drug formulations that may be more easily accepted by patients. Thus, an integrated approach is needed for further improving control, focusing on vulnerable population groups and the local health system.

## Author Summary

Leprosy is still a public health problem in Brazil, and low adherence to multidrug therapy against leprosy (MDT) is an important obstacle of disease control. This may lead to remaining sources of infection, incomplete cure, complications, and multidrug resistance. We performed a study in 78 municipalities in central Brazil, and interviewed leprosy-affected individuals. In total, 3% of patients defaulted, and 18.2% interrupted MDT. Risk factors for interruption of treatment include: reduced number of rooms per household (OR = 1.95; p = 0.04); difficulty in swallowing MDT drugs (OR = 1.66; p = 0.02); temporal non-availability of MDT drugs at health center (OR = 1.67; p = 0.01); and moving residence after diagnosis (OR = 1.58; p = 0.03). Defaulting MDT was significantly associated with: reduced number of rooms per household (OR = 3.43; p = 0.03); moving to another residence (OR = 2.90; p = 0.04); and low family income (OR = 2.42; p = 0.04). Our study shows that defaulting and interruption of MDT against leprosy are associated with some poverty-related variables such as family income, household size, and migration. Intermittent problems of drug supply need to be resolved, mainly on the municipality level. MDT producers should consider drug formulations that are more easily accepted by patients. An integrated approach is needed for further improving control, focusing on most vulnerable population groups and the local health system.

## Introduction

Leprosy control is based on early diagnosis, treatment, and cure, aiming at the elimination of sources of infection and of sequels in affected individuals. Similar to other countries, in Brazil leprosy control measures are integrated into general public health care, thus facilitating access to affected individuals and reduction of disease-related stigma [1].

Interruption and defaulting of multidrug therapy against leprosy (MDT) are still important obstacles of disease control in many endemic countries, with consequences for both patients and the control programs: low adherence is responsible for potentially remaining sources of infection, incomplete cure, and irreversible complications, and in addition may lead to multidrug resistance [2]. In Brazil, the number of patients defaulting treatment was reduced from 3,148 individuals in 2002 to 529 in 2009 (with approximately 49,000 and 37,500 new cases, respectively) [3].

The causes leading to low adherence and non-compliance to MDT are diverse and may include socio-economical, cultural, psychosocial, behavioral, drug-related and disease-related factors, as well as health service-related aspects [2], [4]–[9]. For example, a recent study from India identified stigma as the most common reason given by defaulters, but failed to detail data and to compare these factors with non-defaulters [4]. In Paraíba State in the northeast of Brazil, defaulting of MDT was associated with regular alcohol use, but not with clinical characteristics [5]. However, that study involved only 13 patients who defaulted, as compared to 28 patients finishing treatment regularly. Here we present - as part of a major epidemiological investigation in 78 municipalities in Brazil - population-based data to further investigate factors associated with interruption and defaulting of MDT in a hyperendemic area.

## Methods

### Study area and population

**Figure 1:**
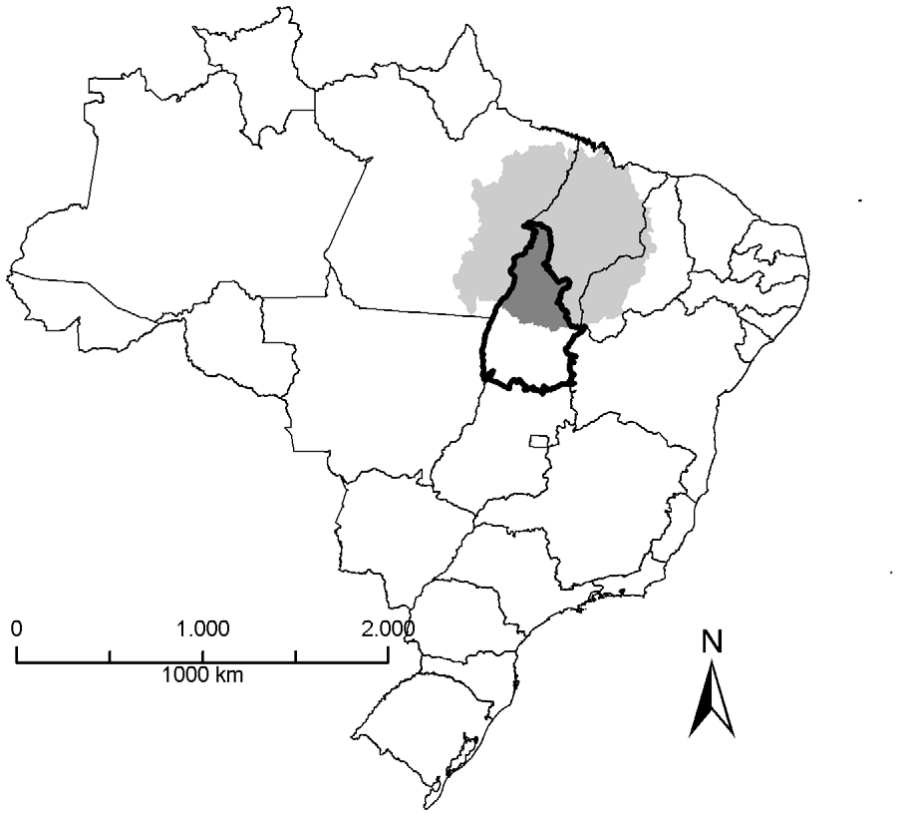
Study area (dark gray area) in Tocantins State, Brazil. The light gray area indicates the cluster of high transmission risk situated in the states Maranhão, Pará, Tocantins and Piauí.

Tocantins State is located in the central savannah region of Brazil (Figure 1). The state has been created in 1988 and has a total population of 1,3 million (2009), distributed throughout 139 municipalities; 83% of the municipalities have less than 10,000 inhabitants. Tocantins is hyperendemic for leprosy: in 2009, a total of 1,345 new cases were notified, and the detection rate was 88.54/100.000 inhabitants.

The present study is part of a major epidemiological investigation performed in 79 municipalities of northern Tocantins. These municipalities are at highest risk for leprosy transmission, according to a recent cluster analysis performed by the Brazilian Ministry of Health (Figure 1) [10], [11]. The target population included all individuals newly diagnosed with leprosy from 2006–2008, living and notified as leprosy cases in these municipalities. We excluded the municipality of Araguaína from the present analysis, the biggest city in the region with about 120 thousand inhabitants. Araguaína has a leprosy reference clinic and shows different characteristics, as compared to the other smaller municipalities that share mainly rural characteristics. These results will be published elsewhere.

We also excluded patients who moved to municipalities outside the endemic cluster, suffered from mental disability or who have shown other characteristics that impeded an interview, such as individuals under the influence of alcohol. Relapsed leprosy cases were also excluded. Individuals who had died after diagnosis were not included in data analysis.

### Study design and data collection

The 78 Municipal Health Secretariats were informed by the Tocantins' State Health Secretariat about the study and the timeframe when the team would perform field visits for data collection. Previous to field visits, the target population was identified in the database of the National Information System for Notifiable Diseases (*Sistema de Informação de Agravos de Notificação* – SINAN). In the municipalities, the patients' charts and the local notification records were first reviewed regarding clinical variables (clinical form, operational classification, disability grade at diagnosis, mode of case detection, date of diagnosis, date of release from treatment and date of last appearance at health center for treatment). If in the local records patients were identified that had not been notified, we included them in the target population. Then, affected individuals were invited by community health agents to be interviewed at the local health care center. If individuals did not present at the health care center, we performed home visits accompanied by local community health agents. Data were obtained at this occasion according to a previously defined framework, using pre-tested structured questionnaires. The framework comprised of four blocks of independent variables possibly associated with the outcomes: 1. Socio-demographic block (gender, age, marital status, education, residence area, number of rooms, number of persons per household, household income, migration); 2. Disease-related block (clinical form of disease, operational classification, disability grade, leprosy reaction, adverse events to MDT, difficulty swallowing MDT drug); 3. Health service-related block (mode of case detection, non-availability of MDT drugs, distance to health care center, perceived difficult access to health care center); 4. Knowledge, attitudes and practices block (alcohol consumption, information of peer persons regarding disease, knowledge on leprosy and cure). Data were collected from September to December 2009.

To reduce inter-observer bias, all questionnaires where applied by two previously trained field investigators (OAC, ARO) who were supervised during the entire study. Data from patients' charts were collected by another two investigators (KH, FW). Extensive pre-tests were performed under supervision.

### Data entry and analysis

Data were entered twice, using Epi Info software version 3.5.1 (Centers for Disease Control and Prevention, Atlanta, USA) and cross-checked for entry-related errors. Answers to open-ended questions were grouped according to similarities and categorized for bivariate analysis. Open-ended questions included information on clinical characteristics for definition of leprosy reaction and adverse events; and questions on knowledge, attitudes and practices. Data analysis was done using STATA version 9 (Stata Corporation, College Station, USA).

As the number of individuals defaulting MDT was relatively low, two separate bivariate analyses were performed, with two different outcomes based on the non-attendance of patients at treatment centers:

1. Defaulting from treatment:
  For this outcome, we used the definition of the Brazilian Ministry of Health [12]: defaulters were defined as individuals that did not complete MDT and who did not present to the health care center for the monthly supervised treatment for at least 12 months. We reviewed the most recently available SINAN database of 2009 regarding this information and in addition collected information on defaulting from the local patients' charts.
2. Interruption of treatment:
  Interruption of MDT was defined as duration of treatment ≥7 months in the case of the paucibacillary form of disease (PB) or ≥13 months in the case of the multibacillary form (MB). Standard MDTs as set by the World Health Organization (and adopted by the Brazilian Ministry of Health) are 6 four-week blister packs for PB, and 12 four-week blister packs for MB patients. Data analysis of interruption included only individuals that had potential time to complete the treatment (all PB cases; MB cases that had begun treatment ≥13 months previous to data collection).

Variables were first analyzed and presented in a bivariate manner. Odds ratios and their respective 95% confidence intervals are given. We applied Fisher's exact test to estimate significance of the difference of relative frequencies. Continuous and discrete variables were not normally distributed and thus compared applying the Wilcoxon rank sum test for unmatched data.

Unconditional logistic regression analysis using backward elimination was then performed to calculate adjusted odds ratios for the independent association between 1) interruption of; and 2) defaulting MDT, and the respective explanatory variables. Results of both analyses are presented separately. In addition to sex, age and leprosy form (PB/MB) which we used as adjusting variables throughout multivariate analysis, variables with a *p* value<0.25 in the Fisher's exact test were entered into the initial regression models, and then backward elimination was run. To remain in the model, a significance of *p*<0.05 was required. Variables were checked for collinearity. Confounding and interaction between variables were also investigated by stratification and by constructing 2×2 tables. All variables that remained in the final models are presented, and odds ratios were adjusted for all other variables in the respective model.

### Ethics

The study was approved by the Ethical Review Board of the Federal University of Ceará (Fortaleza, Brazil) and by the Ethical Review Board of Lutheran University of Palmas (Tocantins, Brazil). Permission to perform the study was also obtained by the Tocantins State Health Secretariat, the State Leprosy Control Program and the municipalities involved.

Informed written consent was obtained from all study participants after explaining the objectives of the study. In the case of minors, consent was obtained from a caretaker. Interviews were always performed separately to guarantee strict privacy, and the diagnosis of leprosy was not given to family members or other community members, in case the patient had not revealed the diagnosis. If any leprosy-associated pathology was observed during the interview or during clinical examination (data of clinical examination to be published elsewhere), participants were referenced to the responsible health care service.

## Results

### Study population and basic characteristics

Of the target population of 1635 individuals from 78 municipalities, 936 (57.2%) from 74 municipalities were included in data analysis; one municipality did not diagnose a single case of leprosy in the study period, and another three municipalities had few cases, but no participants were included (non-consent or not encountered). Twelve patients refused to participate in the study. We excluded another 13 (five under of influence of alcohol that impeded an interview; four convicted; three severely sick who were hospitalized; and one due to advanced age). In addition, 674 were not encountered even after home visits, were not known at the local health centers, or had moved to another city outside the cluster. For the analysis of interruption of MDT 130 individuals were excluded (92 did not have information about date of the beginning of treatment or last date of supervised monthly dose in the health care center, and 38 were classified as MB leprosy with treatment started <13 months before data collection). Thus, data analysis regarding defaulting included 936, and regarding interruption 806 individuals. Information from patients' charts was available in 894 of cases.

Of the total of 936 individuals, 491 (52.5%) were males; the age ranged from 5 to 98 years (mean = 42.1 years; standard deviation: 18.8 years). Two-hundred and twenty-five (24.0%) were illiterate. Median monthly family income was R$ 465 (about 270 USD at the time of the study; interquartile range: R$ 300–R$ 900). In total, 497 (55.6%) were classified as PB leprosy, and 395 (44.1%) as MB.

We identified 28 (3.0%) patients who defaulted MDT; 16 defaulters were included by reviewing the SINAN data information system, and an additional 12 locally in the patients' charts. Only 5 individuals were in the both databases. In total, 147/806 (18.2%) interrupted MDT.

### Factors associated with interruption of MDT

Factors associated with interruption of MDT are detailed in Table 1. Moving to another residence after diagnosis and living in a small residence were significantly associated with interruption. In addition, disease- and health service-related variables (difficulty in swallowing MDT drug; temporal non-availability of MDT drugs) were significantly associated with an increased chance of interruption of treatment (Table 1). Interestingly, disease-related factors such as the clinical form, presence of leprosy reactions or occurrence of adverse events to MDT did not play a significant role.

**Table 1:** Bivariate analysis of factors associated with interruption of, and defaulting multidrug therapy against leprosy.

Variables	Interruption of MDT (n = 806)*				Defaulting MDT (n = 936)*			
	Examined n	Positive n (%)	OR (95% CI)	*P* value	Examined n	Positive n (%)	OR (95% CI)	*P* value
**Socio-demographic**								
Gender								
Male	429	88 (20.1)	1.39 (0.95–2.03)	0.08	491	14 (2.9)	0.90 (0.39–2.07)	0.85
Female	377	59 (15.7)	Reference		445	14 (3.2)	Reference	
Age group (years)								
0–15	67	9 (13.4)	0.70 (0.28–1.60)	0.46	77	3 (3.9)	1.20 (0.19–4.99)	0.60
16–30	181	47 (26.0)	1.59 (0.95–2.67)	0.07	207	7 (3.4)	1.00 (0.30–3.22)	1.00
31–45	205	37 (18.1)	Reference		237	8 (3.4)	Reference	
46–60	200	32 (16.0)	0.86 (0.50–1.50)	0.60	234	8 (3.4)	1.01 (0.33–3.16)	1.00
≥61	153	22 (14.4)	0.76 (0.41–1.40)	0.39	181	2 (1.1)	0.32 (0.03–1.63)	0.20
Marital status								
Single	222	34 (15.3)	0.73 (0.46–1.14)	0.17	256	12 (4.7)	2.20 (0.89–5.43)	0.07
Married	479	95 (19.8)	Reference		549	12 (2.2)	Reference	
Divorced	52	10 (19.2)	0.96 (0.42–2.04)	1.00	63	2 (3.2)	1.47 (0.16–6.82)	0.65
Widowed	52	8 (15.4)	0.73 (0.29–1.65)	0.58	67	2 (3.0)	1.38 (0.15–6.39)	0.66
Education								
Never attended school	191	35 (18.3)	1.00 (0.63–1.55)	1.00	225	6 (2.7)	0.85 (0.28–2.21)	0.83
Attended school at any time	612	112 (18.3)	Reference		707	22 (3.1)	Reference	
Residence area								
Rural	219	45 (20.6)	1.24 (0.82–1.86)	0.30	252	9 (3.6)	1.30 (0.51–3.05)	0.52
Urban	586	101 (17.2)	Reference		683	19 (2.8)	Reference	
Number of rooms per residence								
1–2	55	16 (29.1)	1.95 (0.98–3.70)	0.04	59	5 (8.5)	3.43 (0.98–9.69)	0.03
≥3	749	130 (17.4)	Reference		874	23 (2.6)	Reference	
Number of persons/household								
1–2	25	148 (16.9)	0.90 (0.54–1.47)	0.72	176	9 (5.1)	2.10 (0.82–4.96)	0.08
≥3	657	121 (18.4)	Reference		759	19 (2.5)	Reference	
Household income/month†								
<R$ 465	199	34 (17.1)	0.92 (0.57–1.43)	0.75	232	12 (5.1)	2.42 (1.02–5.63)	0.04
≥R$ 465	545	100 (18.4)	Reference		681	15 (2.2)	Reference	
Moved to another residence after diagnosis								
Yes	179	43 (24.0)	1.58 (1.03–2.40)	0.03	210	11 (5.2)	2.9 (0.95–5.28)	0.04
No	624	104 (16.7)	Reference		722	17 (2.4)	Reference	
**Disease-related**								
Clinical form								
Tuberculoid	148	25 (16.9)	0.91 (0.52–1.60)	0.8	156	9 (5.8)	1.03 (0.29–3.74)	0.6
Boderline	197	38 (19.3)	1.08 (0.65–1.76)	0.8	239	7 (2.9)	3.00 (0.92–10.3)	0.05
Lepromatous	83	14 (16.9)	1.12 (0.54–2.20)	0.9	91	1 (1.1)	0.53 (0.01–4.43)	1.0
Indeterminate	277	50 (18.1)	Reference		290	6 (2.1)	Reference	
Operational classification								
Multibacillary	331	67 (20.2)	1.27 (0.87–1.84)	0.23	496	17 (3.4)	0.74 (0.30–1.72)	0.56
Paucibacillary	473	79 (16.7)	Reference		393	10 (2.5)	Reference	
Disability grade at diagnosis (DG)								
DG II	26	7 (26.9)	1.48 (0.51–3.83)	0.44	–	–	–	–
DG I	134	14 (10.5)	0.47 (0.24–0.87)	0.01	146	4 (10.5)	0.86 (0.20–2.74)	1.00
DG 0	422	84 (19.9)	Reference		471	15 (19.9)	Reference	
Difficulty swallowing MDT drug								
Yes	130	33 (25.4)	1.66 (1.03–2.63)	0.02	153	3 (2.0)	0.60 (0.11–2.01)	0.60
No	671	114 (17.0)	Reference		778	25 (3.2)	Reference	
Type I or II leprosy reaction during treatment (as reported by patient)								
Yes	61	15 (24.6)	1.51 (0.76–2.86)	0.22	75	3 (4.0)	1.39 (026–4.73)	0.49
No	745	132 (17.7)	Reference		861	25 (2.9)	Reference	
Adverse events to MDT (as reported by patient)								
Yes	389	73 (18.8)	1.07 (0.74–1.56)		461	13 (2.8)	0.89 (0.39–2.03)	0.85
No	417	74 (17.8)	Reference	0.72	475	15 (2.9)	Reference	
**Health service-related**								
Mode of case detection at primary health care center								
Spontaneous demand	555	101 (18.2)	Reference		603	20 (3.3)	Reference	
Contact examination	35	5 (14.3)	0.75 (0.22–2.02)	0.66	47	2 (4.3)	1.30 (0.14–5.62)	0.67
Case detection campaign	15	5 (33.3)	2.25 (0.59–7.38)	0.17	157	1 (0.6)	0.19 (0.00–1.19)	0.01
Referred from other center	145	27 (18.6)	1.03 (0.62–1.67)	0.90	18	1 (5.6)	1.71 (0.04–12.07)	0.47
Other	10	1 (10)	0.50 (0.01–3.68)	1.00	10	1 (10)	3.24 (0.70–25.38)	0.30
Temporal non-availability of MDT drug at health care center								
Yes	228	55 (24.1)	1.67 (1.11–2.46)	0.01	265	9 (1.5)	1.19 (0.47–2.82)	0.67
No	573	92 (16.1)	Reference		666	19 (2.9)	Reference	
Distance to health care center								
>30 minutes	154	29 (18.3)	1.04 (0.64–1.65)	0.91	186	5 (2.7)	0.90 (0.26–2.45)	1.00
≤30 minutes	634	116 (18.8)	Reference		731	22 (3.0)	Reference	
Perceived difficult access to health care center								
Yes	172	35 (20.4)	1.18 (0.75–1.84)	0.44	201	3 (1.5)	0.42 (0.81–1.41)	0.17
No	620	110 (17.7)	Reference		721	25 (3.5)	Reference	
**Knowledge and attitudes**								
Continued drinking alcohol during treatment								
Yes	52	14 (26.9)	1.72 (0.83–3.35)	0.10	64	3 (4.7)	0.61 (0.17–3.25)	0.44
No/Never drunk	742	131 (17.7)	Reference		858	25 (2.9)	Reference	
Told household members about leprosy diagnosis								
Yes	778	146 (18.8)	Reference	0.01	907	27 (3.0)	Reference	
No	25	0 (0)	0 (0–0.67)		26	1 (3.9)	1.30 (0.31–8.6)	0.55
Knew leprosy before diagnosis								
Yes	697	121 (17.4)	Reference		808	23 (2.9)	Reference	
No	105	26 (24.8)	1.57 (0.92–2.59)	0.08	124	5 (4.0)	1.43 (0.42–3.96)	0.40
Knew someone with leprosy before diagnosis								
Yes	518	86 (16.6)	Reference		610	16 (2.6)	Reference	
No	282	61 (21.6)	1.39 (0.94–2.03)	0.09	319	12 (4.0)	1.45 (0.62–3.31)	0.41
Thinks that leprosy is curable								
Yes	728	127 (29.0)	Reference		847	25 (3.0)	Reference	
No	38	11 (17.5)	1.92 (0.84–4.14)	0.08	46	3 (6.5)	2.29 (0.43–7.97)	0.17
Does not know	37	8 (21.6)	1.31 (0.50–3.01)	0.51	–	–	–	–

*Information not available in all cases.

**†:** At the time of the survey 1US$ was equivalent to 1.72R$, and R$ 465,- the official minimum wage as set by the Federal Government.

**Figure 2:**
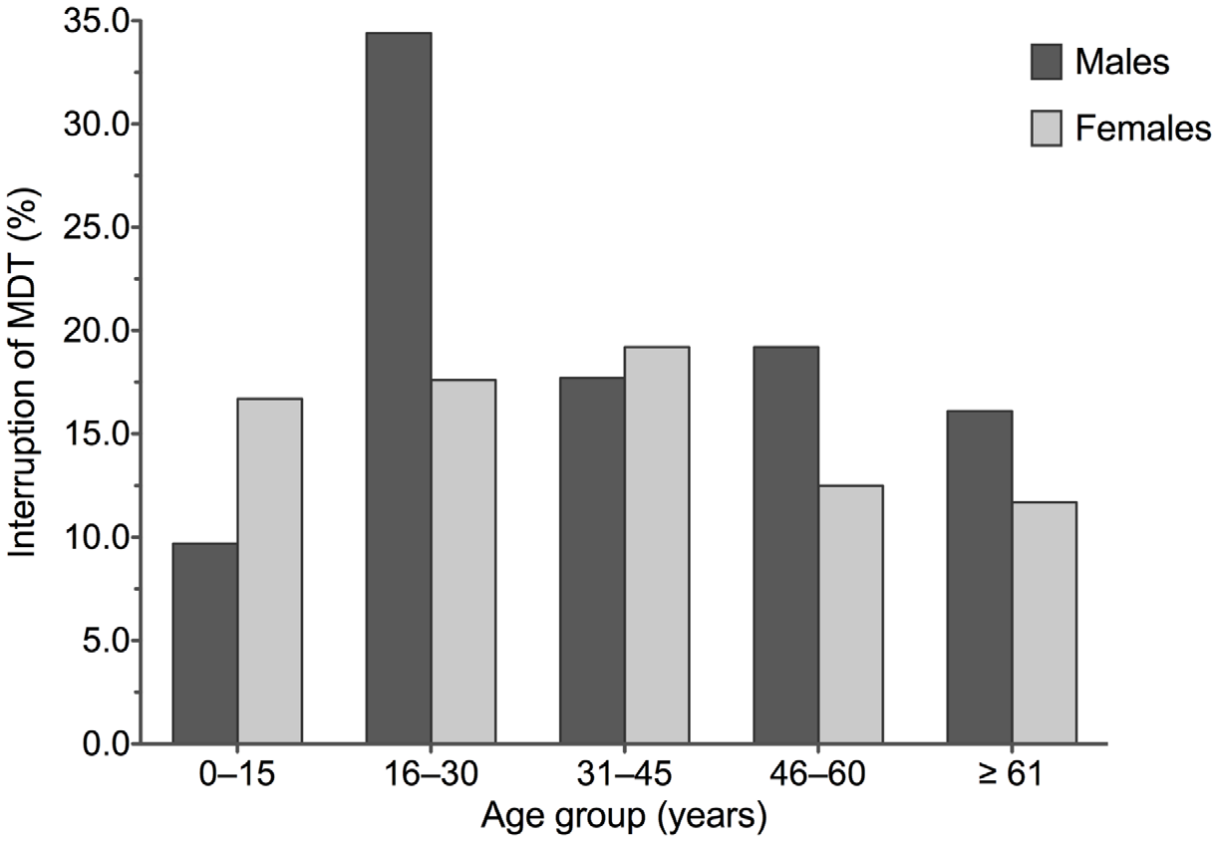
Relative frequency of interruption of MDT, stratified by gender and age group.

Figure 2 depicts the frequency of interruption of MDT, stratified by age groups and gender. In general, the 16–30 year-olds showed the highest chance of interruption, as compared to all other age groups together (OR = 1.84; 95% confidence interval: 1.20–2.77; p = 0.04). This effect could be mainly attributed to the 16–30 year-old males, who showed the highest frequency of interruption (34.4%), roughly a two-fold difference to females of the same age group (17.6%; p = 0.01; Figure 2).

Logistic regression analysis identified temporal non-availability of MDT drugs at the health care center as an independent risk factor for treatment interruption (Table 2). An increased number of rooms per household (as an indicator for wealth) was identified as an independent protective factor.

**Table 2:** Multivariate logistic regression analysis of factors associated with interruption of, and defaulting multidrug therapy against leprosy, adjusted by sex, age and disease classification.

Variables	Interruption of MDT		Defaulting MDT	
	Adjusted OR(95% CI)	*P* value	Adjusted OR(95% CI)	*P* value
Temporal non-availability of drugs at health care center	1.56 (1.05–2.33)	0.03	–	–
Each additional room per residence	0.89 (0.80–0.99)	0.03	0.67 (0.52–0.88)	0.003
Male sex	1.35 (0.93–1.97)	0.12	0.79 (0.36–1.72)	0.55
Age group 16–30 years	0.99 (0.98–1.00)	0.13	1.05 (0.43–2.56)	0.91
Multibacillary disease	1.12 (0.76–1.66)	0.56	0.70 (0.31–1.56)	0.38

### Factors associated with defaulting MDT

Bivariate analysis of factors associated with defaulting MDT is depicted in Table 1. Several socio-economic variables (number of rooms per household; moving to another residence after diagnosis; family income) were significantly associated with defaulting (Table 1). Similar to interruption of MDT, disease-related factors did not play a significant role. Health service variables did also not show any significant association.

In logistic regression analysis, we identified the number of rooms per residence as a factor independently associated with defaulting, with a protective odds ratio of 0.67 for each additional room in the household (Table 2), but no other factors.

## Discussion

Low adherence to drugs is in general a major obstacle in the control of infectious diseases that require prolonged treatment, such as leprosy and tuberculosis. Our comprehensive population-based study shows that poverty, behavior, drug-related and service-related factors were associated with adherence to MDT, hampering leprosy control in a hyperendemic area in Brazil, and suggest evidence-based actions for improving control measures.

It is widely believed that understanding and behavior of patients in relation to drug compliance are largely influenced by their socio-economic condition and level of knowledge; socio-economic factors were previously suggested to influence adherence to MDT [5], [7], [13]. Even though family income as a direct indicator of poverty was not significantly associated with low adherence (but with defaulting), number of rooms was identified as an independent risk factor in both bivariate and multivariable analyses. Poverty and its consequences, similar to other neglected tropical diseases, has been shown to be associated with leprosy in general [14], and our results reflect this complex interaction of causation leading to higher risk of disease in underprivileged populations.

In addition, population movements are usually associated with socio-economic conditions in Brazil. In our study, people who had moved to another residence were more vulnerable for low adherence. These people may lose their bonds with community health workers and other health professionals of the primary health care centers, besides other factors that change in life when moving to another place. Similar findings have been made in India and southeast Brazil, where treatment interruption due to migration has been reported [15], [16]. In the case of tuberculosis, moving to another district with subsequent change of health unit was also shown to increase the risk of defaulting treatment in Uganda [17]. On the other hand, changing residence due to leprosy was clearly not a factor that played a role in our study (data not shown).

Interestingly, the frequency of defaulting MDT was relatively low, as compared to other settings [2], [4], [13], [18], [19], with a rate of only 3%. In Tocantins, the defaulting rate was 47% in 2005, but was reduced drastically in subsequent years [20]. This may reflect the success of efforts made in the last years by Tocantins's health services. In fact, the Brazilian national and state leprosy control programs have put a major effort in improving the decentralized primary health care services, with 90% population coverage of the Family Health Program in Tocantins. As another consequence, variables related to health services seemed to play a minor role for defaulting in our study, despite the identification of temporary shortage of drugs as a significant risk factor for interruption of MDT. We have shown previously that the patients of this area answered most commonly to an open-ended question about the reason for interrupting MDT with temporary shortage of drugs at the health care center, but median time of interruption was only 15 days which indicates that this operational issue was usually resolved quickly [21]. In fact, these logistical problems occurred mainly on the municipality level, as MDT provided by the State Leprosy Control Program to the municipalities did not suffer any shortage in the study period (A.C.F., unpublished observation). In other countries and settings, where leprosy control programs are not yet well established, such as in northern Mozambique, Nigeria and Sudan, health-service related factors play a more crucial role [4], [7], [18], [19], [22].

Our data also indicate that in a setting with an established leprosy control program, clinical variables are of minor importance for low adherence to MDT. In case of leprosy reactions, for example, the primary health care services and the reference centers seem to be prepared to cope with the situation. Similarly, previous studies from northeast Brazil, the Philippines and Nepal suggested that clinical data such as type of leprosy, occurrence of reactions or disability grading at diagnosis would not play a significant role in the given context [2], [5], [23]. Difficulty in swallowing drugs was previously suggested as a factor associated with low adherence to MDT [2]. Considering also the long course of treatment, this shows the need for the search of new formulations that may be better accepted by patients.

Studies from other parts of the world, mainly from the South Asian and Southeast Asian sub-regions, identified other risk factors for low adherence. For example, in the Philippines adverse events were given by the patients as the most important reason (40%) for defaulting [2]. People in Assam (India) who defaulted treatment mentioned loss of occupational hours when going to the health care center (33,1%), adverse events (26,0%) and social stigma (18,1%) as the most common reasons [13]. About 10 years ago, these factors were identified in a qualitative study from Espírito Santo State in Brazil [16]. Since then, Brazilian control programs have improved considerably, e.g. by performing health education on adverse events and leprosy reactions, by training health care professionals and by improved access of the users to the primary health care system. The results of our study reflect these efforts and highlight the differing situation in other countries.

Available evidence on the influence of demographic variables on adherence to treatment is contradictory. Similar to the study from the Philippines [2], demographic data such as gender, age and civil status were not associated with low adherence in our study population. In contrast, in endemic regions of Nepal and India, more males than females completed treatment, and illiteracy was also significantly associated with low treatment compliance [9], [13]. However, both studies had some methodological problems, and analysis of data is limited. Interestingly, our study showed highest interruption rates in young males, when data were stratified by gender. This indicates that factors are multifaceted and that in this case, young males, who are generally known to show insufficient health care behavior, should be considered a vulnerable group for low adherence. In fact, the Brazilian Ministry of Health has taken into consideration the special needs of the male population and recently launched an integrative program focusing on male gender issues [24].

Similar to leprosy, tuberculosis needs prolonged treatment and has also shown to reveal problems regarding adherence. Improving adherence to treatment against leprosy can thus be expected to have positive impact also on other diseases, such as tuberculosis. In fact, the factors associated with low adherence to tuberculosis are similar. For example, in Ethiopia, the occurrence of adverse events to tuberculosis treatment was found to be a significant risk factor for defaulting, whereas knowledge about duration of treatment was protective and increased the odds of terminating treatment [25]. A study from Nepal identified distance to health care services and low knowledge on disease and its treatment as risk factors for non-adherence to tuberculosis directly observed short-course (DOTS) [26].

An ancillary finding was the detection of incomplete patients' charts and registries in many cases. We detected in total 128 leprosy cases that were not included in the national SINAN database for notifiable diseases, and a considerable number of cases of abandonment from treatment, which had not been registered as such in SINAN. In addition, only in 72.1% (645/894) information on degree of disability at diagnosis was available in the patients' charts. The quality of patients' records and datasets has improved in the past years, but there is still a clear need for more complete data sets and patient charts, as suggested recently in a study performed in northeast Brazil [27].

Though being a population-based study performed in a considerable number of municipalities in a leprosy hyperendemic region, our study is subject to limitations. First, the number of defaulters, as a result of the ongoing leprosy control measures, has been reduced significantly in the past years, and we included only 28 patients who defaulted treatment. This hampered statistical analysis to some degree. Second, non-participation bias, mainly of those who abandoned treatment, may have played a role. Thus, we performed an additional analysis using a less stringent criterion for compliance: interruption of treatment, based on the duration of treatment. However, this analysis did not take into account adherence to drugs taken at home, but was based on appearance at the health care centers for the monthly supervised dose, which should be taken into account in the interpretation of results. Finally, incomplete patients' charts and subsequent missing data hampered analysis regarding clinical variables in some cases. On the other hand, integration of local primary health care professionals and of the State and Municipal Leprosy Control Programs reduced non-participation bias.

We conclude that in an area in Brazil where leprosy control actions are well established, adherence to MDT is a result of a complex interaction between different socio-cultural, service-related, drug-related and economical factors. Intermittent problems of drug supply need to be resolved, mainly on the municipality level. MDT producers should consider oral drug formulations that may be more easily accepted by patients. An integrated approach is needed to further improve adherence and other aspects of leprosy control, such as early diagnosis, including the stakeholders involved: patients and their families, health care professionals, and policy makers [6], [28], [29]. Improved adherence to MDT will further improve the leprosy control programs and in addition minimize the risk of possibly upcoming drug resistance.

## References

[1] Souza AD, el-Azhary RA, Foss NT (2009). Management of chronic diseases: an overview of the Brazilian governmental leprosy program.. Int J Dermatol.

[2] Honrado ER, Tallo V, Balis AC, Chan GP, Cho SN (2008). Noncompliance with the world health organization-multidrug therapy among leprosy patients in Cebu, Philippines: its causes and implications on the leprosy control program.. Dermatol Clin.

[3] Anonymous (2010). Hanseníase - casos confirmados notificados no Sistema de Informação de Agravos de Notificação - Sinan Net.. http://dtr2004.saude.gov.br/sinanweb/tabnet/dh?sinannet/hanseniase/bases/Hansbrnet.def.

[4] Rao PS (2008). A study on non-adherence to MDT among leprosy patients.. Indian J Lepr.

[5] Trindade LC, Zamora AR, Mendes MS, Campos GP, Pontes de Aquino JA (2009). Factors associated with non-adherence to leprosy treatment in João Pessa, Paraíba State (Brazil).. Cadernos Saúde Coletiva.

[6] Williams MC (2005). How can adherence with multi-drug therapy in leprosy be improved?. Lepr Rev.

[7] Heijnders ML (2004). An exploration of the views of people with leprosy in Nepal concerning the quality of leprosy services and their impact on adherence behaviour.. Lepr Rev.

[8] el Hassan LA, Khalil EA, el-Hassan AM (2002). Socio-cultural aspects of leprosy among the Masalit and Hawsa tribes in the Sudan.. Lepr Rev.

[9] Kumar RB, Singhasivanon P, Sherchand JB, Mahaisavariya P, Kaewkungwal J (2004). Gender differences in epidemiological factors associated with treatment completion status of leprosy patients in the most hyperendemic district of Nepal.. Southeast Asian J Trop Med Public Health.

[10] Penna ML, de Oliveira ML, Penna GO (2009). The epidemiological behaviour of leprosy in Brazil.. Lepr Rev.

[11] Penna ML, Wand-Del-Rey-de-Oliveira ML, Penna G (2009). Spatial distribution of leprosy in the Amazon region of Brazil.. Emerg Infect Dis.

[12] Anonymous (2002). Guia para o Controle da Hanseníase.

[13] Kar S, Pal R, Bharati DR (2010). Understanding non-compliance with WHO-multidrug therapy among leprosy patients in Assam, India.. Journal of Neurosciences in Rural Practice.

[14] Kerr-Pontes LR, Barreto ML, Evangelista CM, Rodrigues LC, Heukelbach J (2006). Socioeconomic, environmental, and behavioural risk factors for leprosy in North-east Brazil: results of a case-control study.. Int J Epidemiol.

[15] Naik SS, More PR (1996). The pattern of ‘drop-out’ of smear-positive cases at an urban leprosy centre.. Indian J Lepr.

[16] Fogos AR, Araújo Oliveira ER, Teixeira Garcia ML (2000). Analysis of reasons for treatment drop out - the case of hansen's disease patient of the Health Unit at Carapina/ES.. Hansenologia Internationalis.

[17] Nuwaha F (1997). Factors influencing completion of treatment among tuberculosis patients in Mbarara District, Uganda.. East Afr Med J.

[18] Griffiths S, Ready N (2001). Defaulting patterns in a provincial leprosy control programme in Northern Mozambique.. Lepr Rev.

[19] Coebergh JA, Buddingh H (2004). Non-adherence to leprosy treatment in Western Sudan; the people behind the numbers.. Lepr Rev.

[20] Anonymous (2005). IV Carta de eliminação da hanseníase - Tocantins, 2005.

[21] Chichava OA, Ariza L, Oliveira AR, Ferreira AC, Marques da Silva LF (2011). Reasons for interrupting multidrug therapy against leprosy: the patients' point of view.. Lepr Rev.

[22] Nwosu MC, Nwosu SN (2002). Leprosy control in the post leprosaria abolition years in Nigeria: reasons for default and irregular attendance at treatment centres.. West Afr Med J.

[23] Chalise SC (2005). Leprosy disease in Nepal: Knowledge and non-compliance of patients.. J Nep Med Assoc.

[24] Anonymous (2008). Política nacional de atenção integral à saúde do homem (princípios e diretrizes).

[25] Tekle B, Mariam DH, Ali A (2002). Defaulting from DOTS and its determinants in three districts of Arsi Zone in Ethiopia.. Int J Tuberc Lung Dis.

[26] Wares DF, Singh S, Acharya AK, Dangi R (2003). Non-adherence to tuberculosis treatment in the eastern Tarai of Nepal.. Int J Tuberc Lung Dis.

[27] Galvao PR, Ferreira AT, Maciel MD, De Almeida RP, Hinders D (2008). An evaluation of the Sinan health information system as used by the Hansen's disease control programme, Pernambuco State, Brazil.. Lepr Rev.

[28] Siddiqui MR, Velidi NR, Pati S, Rath N, Kanungo AK (2009). Integration of leprosy elimination into primary health care in orissa, India.. PLoS ONE.

[29] Lockwood DN, Suneetha S (2005). Leprosy: too complex a disease for a simple elimination paradigm.. Bull World Health Organ.

